# Relapsed Mpox Keratitis, St. Louis, Missouri, USA

**DOI:** 10.3201/eid3007.240388

**Published:** 2024-07

**Authors:** Cinthia Pi, Osasu Adah, Preetam A. Cholli, Roosecelis Martines, Getahun Abate, Lori Hainaut, Erich Seipel, T. Scott Isbell, Roddy Frankel, Nongnooch Poowanawittayakom

**Affiliations:** St. Louis University, St. Louis, Missouri, USA (C. Pi, O. Adah, G. Abate, L. Hainaut, E. Seipel, T.S. Isbell, R. Frankel, N. Poowanawittayakom);; CDC, Atlanta, Georgia, USA (P.A. Cholli, R. Martines)

**Keywords:** ocular mpox, mpox keratitis, ocular syphilis, HIV, AIDS, monkeypox, virus, viral infection, Missouri, United States

## Abstract

We describe a case of a 46-year-old man in Missouri, USA, with newly diagnosed advanced HIV and PCR-confirmed mpox keratitis. The keratitis initially resolved after intravenous tecovirimat and penicillin for suspected ocular syphilis coinfection. Despite a confirmatory negative PCR, he developed relapsed, ipsilateral PCR-positive keratitis and severe ocular mpox requiring corneal transplant.

Ocular mpox, caused by monkeypox virus (MPXV), can threaten eyesight and requires prompt ophthalmologic consultation. Understanding of the disease course, treatments, and treatment durations is limited. We describe a case of relapsed mpox keratitis after an initial PCR-confirmed resolution. The patient provided informed consent for this report’s publication.

## The Case Study

**Figure 1:**
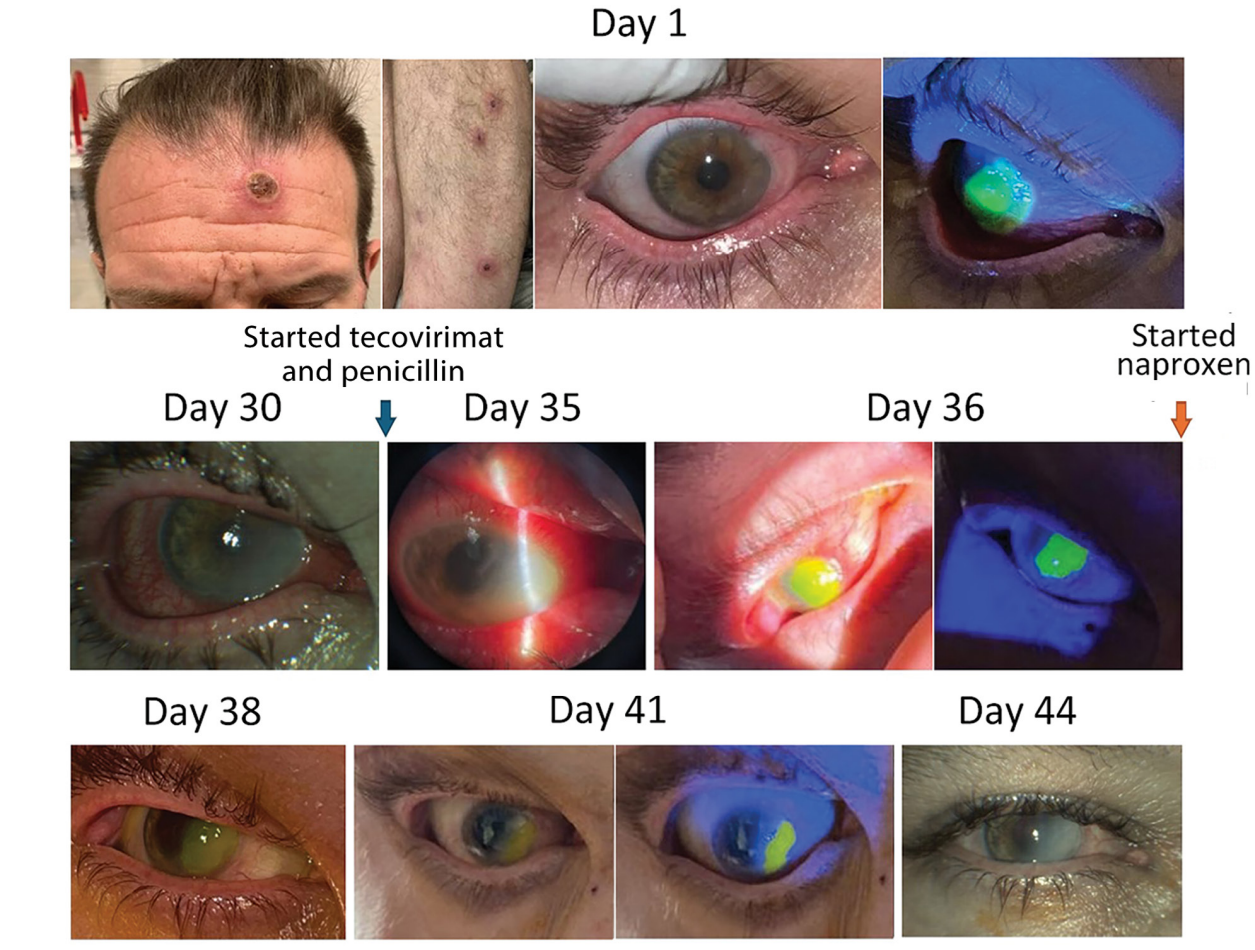
Physical and ophthalmologic examination results over time of a 46-year-old patient in St. Louis, Missouri, USA, after receiving mpox and HIV diagnoses. The exam remained unchanged after day 44 despite treatment. IV, intravenous.

A 46-year-old man sought care at St. Louis University Hospital in St. Louis, Missouri, USA, after 1 month of ulcerating skin lesions and a painful right corneal ulcer. He reported changing his monthly soft contact lenses every 2–3 months and having unprotected sex with multiple male partners. An eye examination showed diminished right eye visual acuity (20/70; baseline unknown), and the right cornea had a large (8.5 × 7.3mm) ovoid ring extending onto the nasal bulbar conjunctiva with central infiltrate and an overlying epithelial defect (Figure 1). Laboratory testing for HIV-1 antibody was reactive; viral load was 1.1 million copies/mL and absolute CD4 count 75 cells/μL (reference range 430–1,800 cells/μL). We conducted PCR testing from biopsies of the patient’s skin lesions and corneal swabs and detected nonvariola orthopoxvirus DNA consistent with MPXV. Laboratory testing for other sexually transmitted infections returned *Treponema pallidum* antibody and particle agglutination positive, rapid plasma reagin nonreactive. Hepatitis C, B, and urine gonorrhea/chlamydia tests were negative. Treatment for possible ocular syphilis was deferred because of discordant syphilis test results and the positive orthopoxvirus PCR. 

We started the patient on oral tecovirimat and bictegravir/emtricitabine/tenofovir alafenamide for HIV. The patient’s vision and examination results remained unchanged, but he reported improved eye pain. He was discharged on day 7 of hospitalization with a regimen of bictegravir/emtricitabine/tenofovir, trimethoprim/sulfamethoxazole, and oral tecovirimat for 2 weeks.

Two weeks after completing oral tecovirimat (29 days after the initial visit), the patient returned for recurrent right eye pain. His skin lesions had fully resolved. An eye examination showed unchanged visual acuity and epithelial defect (8.5 × 7.3mm). The patient was readmitted, and the Center for Disease Control and Prevention’s mpox clinical consultations team was contacted.

We initiated treatment with intravenous (IV) tecovirimat, topical trifluridine eye drops, antibiotic eye drops, 2% cyclopentolate, and oral analgesics. Laboratory testing showed improved CD4 count (336 cells/μL) and decreased HIV viral load (430 copies/mL). Repeat corneal PCR of the right eye remained orthopoxvirus positive. Ocular herpes simplex virus 1 and 2 PCR and varicella zoster virus PCR results were negative. Serum cytomegalovirus PCR showed low-level detection (<50 IU/mL).

Despite treatment, the patient’s keratopathy worsened. We began empiric treatment for ocular syphilitic scleritis with a 14-day course of IV penicillin. The patient reported significant pain improvement, and examination of his eye showed a decreasing corneal ulcer (5 × 5 mm) with completely healed conjunctival epithelium, suggesting the patient possibly had ocular syphilis. We transitioned the patient to oral tecovirimat on day 8 and discharged him on day 12 of readmission with a 1-week course of prednisolone acetate eye drops (4×/d) and a 14-day course of oral tecovirimat.

At 1-week follow-up, the patient’s visual acuity had improved to 20/30, and all ocular symptoms resolved except for a 1.7 × 4.5 mm epithelial defect. Results of repeat MPXV PCR of the right eye lesion were negative. Repeat absolute CD4 was 365 cells/μL and HIV viral load was 191 copies/mL.

Eighty-five days after the patient initially sought care, he returned with relapsed right eye pain. Examination revealed worsening visual acuity to 20/300 with a new right corneal ulcer overlapping the site of a previous fully healed scarred lesion. We ordered a repeat MPXV PCR that was positive. The patient denied interval sexual partners, known mpox exposures, or other mpox symptoms; we interpreted findings as evidence of relapsed mpox keratitis.

The patient declined brincidofovir or additional tecovirimat to avoid side effects, including dry skin with prior tecovirimat, but was amenable to monthly ophthalmology follow-up. His eye pain and keratitis resolved over serial visits, but he had residual corneal infiltrates requiring corneal transplant. Surgery was performed, and his postoperative course was uneventful.

**Figure 2:**
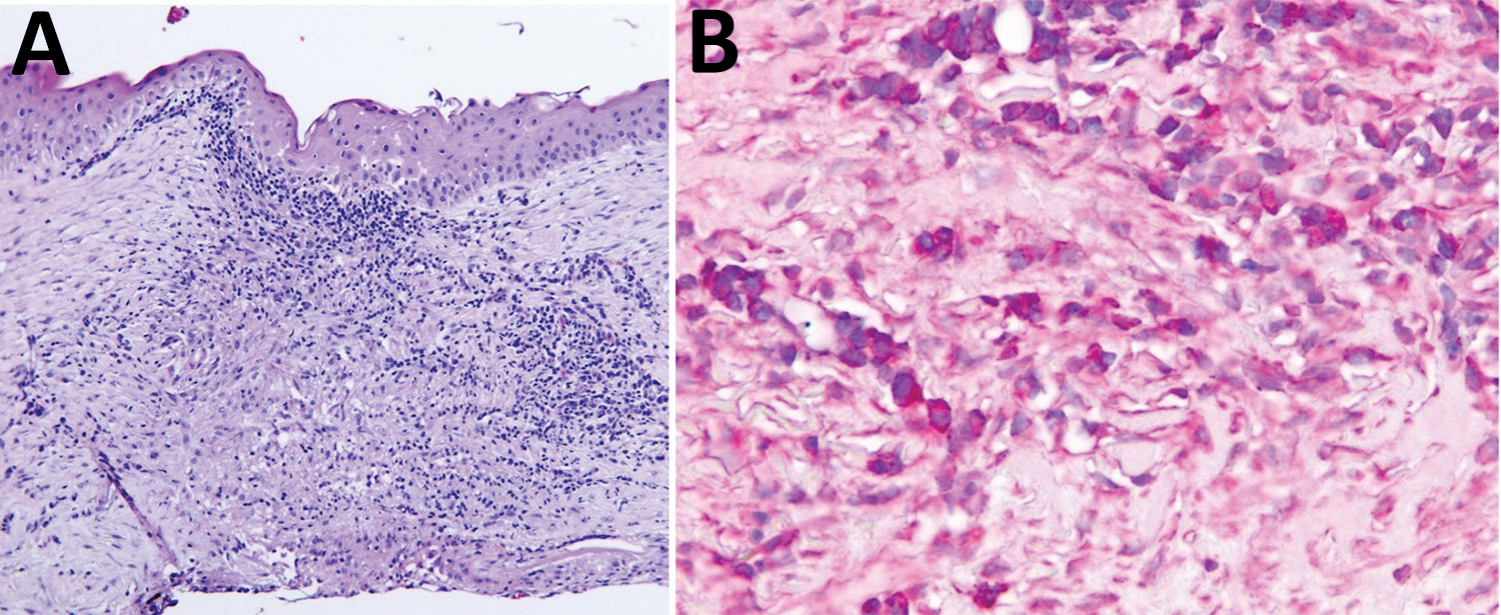
Right eye cornea histopathology from a 46-year-old patient in St. Louis, Missouri, USA, with ocular mpox diagnosis. Sample was collected on day 217 after the patient initially sought care. A) Corneal epithelium is intact. Stromal lesion shows focal scarring with inflammation composed of lymphocytes, plasma cells, and rare neutrophils. B) Orthopoxvirus antigens detected by immunohistochemistry in area of stromal lesion.

We conducted a corneal biopsy and found stromal scarring with vascular proliferation and mixed inflammatory infiltrate with lymphocytes, plasma cells, and rare neutrophils. No cytoplasmic inclusion bodies were observed. We conducted an immunohistochemistry assay by using a MPXV polyclonal rabbit antibody and orthopoxviral antigens were identified in the stromal mononuclear cells (Figure 2 panels A, B). The patient had a dense cataract in the same eye (previously obscured by the corneal opacity) and underwent cataract surgery 4 months after the corneal transplant; results of orthopoxvirus PCR of the aspirated lens material were negative.

PCR is highly sensitive for detecting MPXV in conjunctival and corneal samples (*1*–*4*). Surface PCR tests of this patient’s corneal ulcer remained positive after 1 round of antiviral treatment, despite restored immunity and ongoing antiretroviral therapy. His protracted PCR positivity could have been be because of residual mpox DNA rather than active viral replication. Treatment decisions were based on his corneal lesions and pain symptoms. However, PCR results correlated well with his symptoms. His PCR results became negative 2 weeks after resolution of his initial keratitis.

Our patient developed PCR-confirmed mpox keratitis over his healed lesion >40 days after his initial episode resolved. The identical location and lack of systemic mpox symptoms raised concerns for possible recrudescence from a persistent subclinical viral reservoir. Whereas the negative PCR could have been a false negative, PCR samples were all obtained in the same manner, and specimen collection was not considered to have been a factor in the test results. PCR testing was positive from corneal ulcer swab samples and from within the patient’s excised cornea but not from within aspirated lens material from his subsequent cataract surgery. This result suggests the infection was restricted to the ocular surface and did not penetrate the anterior chamber or lens.

There is 1 reported case of recrudescent corneal erosion from cowpox (a related orthopoxvirus), attributed to viral persistence in the presence of systemic immunosuppressants and steroid eye drops (*5*). In the case we report, the patient’s steroid drops were discontinued >1 month before his relapsed keratitis. An external viral reservoir could have been a source for reinfection, although our patient’s relapse occurred beyond the windows in which MPXV persists on surfaces at ambient temperatures in environmental and experimental contexts (*6*–*8*).

Prevalence of HIV and STI co-infection in the recent mpox outbreak has been high; an international case series found 41% concurrent HIV and 9% concurrent syphilis infections in confirmed cases of mpox (*9*). The patient we report had newly diagnosed advanced HIV, and his first corneal ulcer resolved after he began empiric penicillin for possible ocular syphilis. It remains unclear if his initial keratitis resolved due to the natural course of illness, restored native immunity from antiretroviral therapy, transitioning from oral to IV tecovirimat that may have enabled more reliable tecovirimat absorption, or cotreatment for syphilitic sclerokeratitis. 

## Conclusions

This case highlights the importance of considering ocular co-infections, particularly in cases of persistent or prolonged mpox. Systemic treatment should be considered for all ocular mpox cases (*10*). However, the efficacy of options remains unclear (*10*), and there are no data regarding ocular penetration of oral versus IV tecovirimat. Our understanding of ocular mpox treatment comes only from case reports. Patients with ocular infections need close follow-up. Immunocompromised patients may have more frequent and unusual complications than normal hosts and may require prolonged (>14 days) courses of tecovirimat until native immunity is restored (*11*).
